# Study of the impact on emissions and engine performance of diesel fuel additives made from cotton and castor blended seed oils

**DOI:** 10.1016/j.heliyon.2025.e41659

**Published:** 2025-01-02

**Authors:** Hailegebrel Zewdie Woldetensy, Dinku Seyoum Zeleke, Getachew Shunki Tibba

**Affiliations:** Department of Mechanical Engineering, Addis Ababa Science and Technology University, Addis Ababa, 16417, Ethiopia

**Keywords:** Bioadditives, Engine performance & emission, Diesel fuel, Internal combustion engine

## Abstract

Many approaches have been implemented in order to reduce the emissions of particular pollutants without compromising engine performance. Cotton and castor mixed seed oil was chosen for the current study due to their distinct fatty acid composition and potential as a feedstock for bio-additives. Three fuel samples—99 % diesel and 1 % blended fuel (cottonseed oil + castor seed oil), 99.50 % diesel and 0.50 % (cottonseed oil + castor seed oil) blended fuel, and 100 % diesel fuel—are examined. Gas chromatography was used to assess the fatty acid makeup of the substances under investigation. A TBMC8 test bench was used to measure the performance and exhaust emissions characteristics of the diesel fuel containing additives of cotton and castor seed oil. Brake-specific fuel consumption (BSFC), brake thermal efficiency (BTE), engine torque, and emission characteristics of the diesel with additives are measured by adjusting an engine load at 0 %, 20 %, 40 %, 60 %, and 80 %. For D99 (cottonseed oil + castor seed oil) 1, BSFC, BTE, and engine torque at 20 % engine load are 0.757 kg/kWh, 32.98 %, and increased by 1.1 %, respectively. When engine load increased, BSFC slightly increased by 1.1 %. Unlikely, as an engine load increases, there is a modest drop in both BTE and engine torque. Due to the increased oxygen content of bio-additives, which aids in CO oxidization during combustion, carbon monoxide (CO) emissions have dropped by 1.5 % for engine loads ranging from 0 % to 80 %. The higher oxygen content of biodiesel significantly reduced CO emissions, however higher oxygen percentages in blends of biodiesel led to a rise in CO2 emissions. Because cotton and castor blended additives ignited more quickly, NOx increased. Nevertheless, all fall within the allowed range of the ASTM standard.

## 1 Introduction

Diesel engines have been instrumental in the advancement of human technology. Since the days of Rudolf Diesel, these have been the most effective tools for converting heat energy into mechanical energy. It supplies enough energy for power production, transportation, agriculture, and other uses. However, the usage of diesel fuel has been linked to other environmental issues, such as climate change and air pollution. Diesel engine exhaust will produce a significant amount of oxides due to the high sulphur content of common fossil diesel fuel used in engines. The toxic oxides can produce acid rain and air pollution, both of which are detrimental to human health [[1], [2], [3]]. The removal of SOx has emerged as the most urgent issue in the field of air pollution control as SOx pollution has become a more serious worldwide concern.

Diesel fuel loses some of its lubricity when polar compounds like heteroatoms and polyaromatics are removed during the desulfurization process. These chemicals create a protective coating or lubricating film between the metal mating surfaces to reduce wear and friction. Furthermore, ultra-low sulphur diesel's lubricity will be significantly decreased by eliminating polar molecules [4]. Diesel fuel's lubricity is crucial for engine components since it primarily lubricates injection system items including injection pumps [5]. Recently, there has been a lot of interest in vegetable oil additives for diesel fuel due to their potential to improve engine efficiency and lower hazardous emissions. Low-sulphur diesel fuel has been enhanced with vegetable oils to increase its lubricating capabilities. Compared to diesel fuel additives based on minerals, they are more environmentally beneficial due to their renewable nature and biodegradability [6].

Vegetable oil is widely accessible across the globe. Unlike petroleum-derived fuels, which have a carbon cycle that lasts millions of years, this renewable fuel has a short carbon cycle of 1–2 years and is environmentally friendly. Worldwide study is being prompted by these to examine vegetable oils and their byproducts as potential substitutes for petroleum-based fuel. Different pressing techniques, solvent extraction, or mixes of these are used to extract vegetable oils from oil-bearing seeds, fruits, or nuts [7]. Nonetheless, a significant drawback of vegetable oil is its viscosity, which is ten times more than that of mineral diesel. Moreover, biodiesel produced from vegetable oil is known to have poorer oxidation stability than fuels derived from petroleum [8]. Poor fuel atomization from the high viscosity of the vegetable oil can result in poor combustion, ring sticking, injector cocking, deposits in the injector, injection pump failure, and lubricating oil dilution due to crankcase polymerization [9,10]. On the other hand, there are benefits to the lubricity of vegetable oil additions. For instance, it can reduce engine friction losses, improving engine efficiency and performance [11,12]. At room temperature, vegetable oils are not suitable for direct use in diesel engines. To improve engine performance, the viscosity of vegetable oils must be reduced. There are three effective methods for lowering the viscosity of vegetable oils: heating, mixing with a lighter oil, and transesterification [8,13]. A minimum of 1 % (10000 ppm) or even 2 % (20000 ppm) of biodiesel is usually needed to improve lubricity.

Furthermore, the cost of raw materials, which accounts for 70–85 % of the entire production cost, is still expensive for the components of biodiesel blends [[14], [15], [16], [17], [18], [19], [20]]. The cost is now the main barrier preventing the large-scale production of lubrication blend components (such as biodiesel). For ULSD fuels, it is crucial to find raw materials that can be used to increase the lubricity of the fuel by using effective additives at low additive levels. Using the mix approach, one can easily, economically, and efficiently reduce the viscosity of pure vegetable oil. To attain engine performance similar to diesel fuel, straight vegetable oil can be blended with low-viscosity fuels like alcohol and diesel to reduce viscosity [10].

Depending on the feedstock oil or fat utilized, the final biodiesel will have a different position in the trade-off between cetane number, oxidation stability, and cold flow ability. Higher cetane levels and lower cold flow characteristics are observed in biodiesel produced from more saturated feedstock, likely due to enhanced oxidation stability. The cold flow properties of biodiesel produced from feedstock oil or fat with low saturated content are superior, despite its lower cetane number and worse oxidation stability. The more polyunsaturated portion of cottonseed oil and the more ricinoleic acid (18:1-OH) component of caster seed oil were mixed to obtain the best bio additive properties. Studies specifically investigating the impacts of blending castor and cottonseed oil are still limited, despite prior research on the effects of biodiesel blends on engine efficiency and emissions. Ethiopia is one of several African countries that produce and export cotton. It has a long history of cotton farming, with an estimated 2.6 million hectares suitable for this crop. Of these, 65 % are in high potential cotton-producing areas, with the remaining 0.9 million hectares, or 35 %, in medium potential districts. Of the total land under cotton cultivation, 33 % is cultivated by smallholders, 45 % by private farms, and 22 % by state-owned farms [18].

This study examined into how diesel fuel additives made of mixed seed oils—cotton and castor—affect engine performance and emissions. Bio-additives have the potential to enhance the sustainability and efficiency of diesel engines, hence offering valuable insights into the benefits and possible risks associated with the use of these particular bio-additives in diesel fuel blends. Characterization revealed that the produced crude and refined cotton and castor oil satisfied all assessed characteristics (specific gravity, refractive index, acid value, saponification value, and iodine value) in accordance with ASTM standard requirements.

## 2 Materials and methods

### 2.1 Preparation of diesel fuel with bio-additives

Determining whether a one-step or two-step preparation procedure is necessary depending on FFA content is the first stage in the production of biodiesel. The FFA content was computed using the raw oil's acid number. The triglyceride content's increased FFA content results in a significant loss of product yield and saponification formation. As a result, there are two steps in the conversion process. When the oil generated from the feedstock has higher FFAs (>1%w/w), such as castor and cottonseed oils. The first step is esterification, a pre-treatment method that uses acid catalysts to remove contaminants. The final product is then subjected to base-catalyzed transesterification. For the experiment, a 500 mL three-neck round bottom flask, a mechanical stirrer, and a reflux system were employed in a laboratory-scale reactor. In the reactor, 200 mL of castor seed oil and 72 mL of methanol were combined at a molar ratio of 9:1. In the esterification process, H2SO4 was used as the acid catalyst. It was measured at 1 % (w/w) of the pre-heated oil and maintained at 60 °C with constant stirring at 500 rpm. For 2 h, the reaction mixture was allowed to react in the reactor flask. Following the esterification procedure, two separate liquid layers were visible in the esterified oil sample that had been let to settle in a separating funnel for a full day. While the lower phase— esterified oil — was utilized for the second stage of transesterification, three rounds of distilled water washing eliminated the top layer, which contained methanol and catalyst residues.

**Fig. 1:**
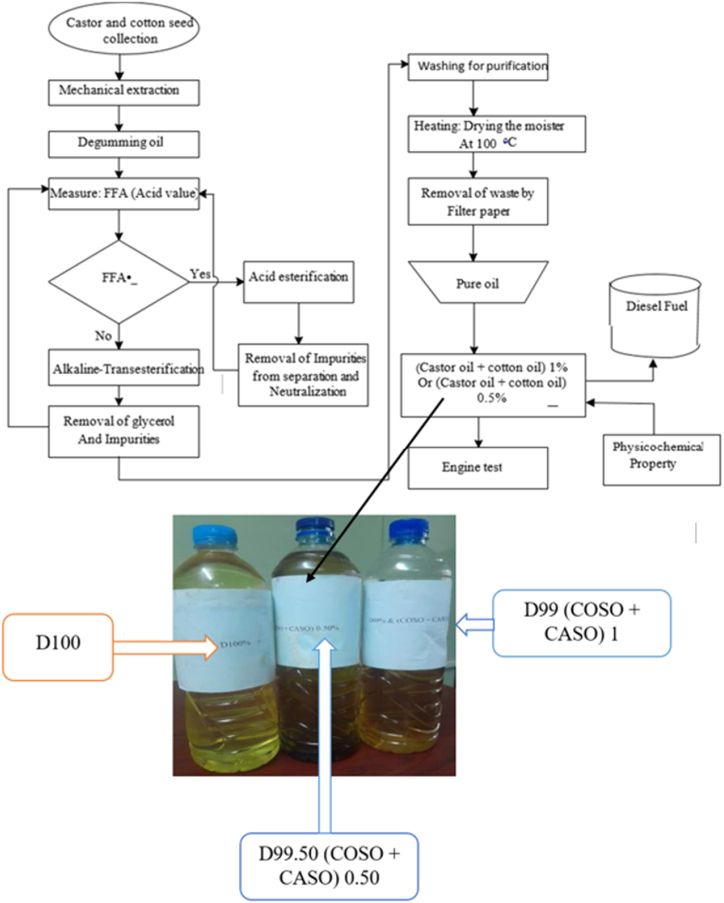
The extraction process and tested fuel sample.

The entire extraction procedure and the impact of blending bio-additives on diesel fuel are depicted in Fig. 1. D99.50 (COSO + CASO) 0.50 (99.50 % Diesel + 0.50 % Bio-additives (cottonseed oil with castor seed oil blend)), D99 (COSO + CASO) 1 (99 % Diesel + 1.00 % Bio-additives (cottonseed oil with caster seed oil blend), and D100 (100 % Diesel + 0 % Bio-additives +) were the three different fuels that were prepared, as Fig. 1 illustrates. For this investigation, fuel samples were made by doping or adding additives of castor seed and blended cotton oil to diesel fuel. The produced fuel samples were compared to diesel fuel that is sold commercially (D100). Diesel and a blend of cotton and castor oil were mixed to generate D99.50, which had a 0.50 mixture of additives. After mixing 99.50 % diesel (by volume) with a mixture of 0.50 % cotton and castor oil, the mixture was homogenized after 30 min at 700 rpm. Similar procedures are repeated for D99 (COSO + CASO) 1 (99 % Diesel + 1.00 % bio-additives (cottonseed oil with caster seed oil blend). The blended gasoline is then left in its homogeneity without sedimentation for a period of two weeks.

Castor and cottonseed oil biodiesel's physicochemical properties, such as its density, kinematic viscosity, calorific value, and total sulphur content, were measured both with and without diesel. Comparably, the energy content of the fuel is indicated by the calorific value, commonly referred to as the heating value. A lower calorific value of biodiesel than petroleum diesel may affect engine performance. When compared to petroleum fuel, cottonseed and castor biodiesel are typically better at reducing the sulphur content. Using the ASTM criterion, the physicochemical characteristics of the diesel fuels and bio-additives employed in the study were investigated in the chemistry laboratory of Addis Ababa Science and Technology University and the Ethiopian Petroleum Institute. The results of the test is depicted in Table 1.

**Table 1:** Physicochemical properties of tested fuel samples [2].

Property	Test Methods	Physicochemical properties of fuel and its additives		
		Diesel fuel	D99.50 % + 0.50 % (cotton &Caster seed oil)	D99.00 % + 1.00 % (cotton &Caster seed oil)
Density@15 °C, kg/m^3^	D4052	843.4000	844.2000	845.0000
Density@20 °C,kg/m^3^	D4052	839.9000	840.7000	841.5000
Kinematic viscosity 40 °C mm^2^/s	D445	3.004	3.0319	3.0600
Calorific value, MJ/kg	Calculated	45.5416	45.5249	45.5081
Total sulphur, %WT	D4294	0.0484	0.0482	0.0481

With gas chromatography (Agilent 7890B) and a mass spectroscopy detector (Agilent 5977A MSD, USA), the FA composition of cottonseed oil, castor oil, and cottonseed oil (50 %) coupled with castor seed oil (50 %) was determined using the European standard (EN 14103:2011). The dimensions of the HP-88 type column are 30 mm in length, 0.25 mm in internal diameter, and 0.20 μm in film thickness. Table 2 displays the fatty acid composition of castor oil, cottonseed oil, and 50 % cottonseed oil combined with 50 % castor seed oil.

**Table 2:** Fatty acid composition (wt, %) of cottonseed oil, castor seed oil, and cottonseed oil blend with castor seed oil [2].

Fatty Acid Name	Chemical Structure	Cottonseed Oil (%)	Castor seed oil (%)	Cotton seed Oil blend with Castor seed oil (%)
Methyl 8,9-octadecadienoate (Linoleic acid,C18:2)	C_19_ H_34_ O_2_	52.9455		49.9018
Z-(13,14-Epoxy)tetradec-11-en-1-ol acetate	C_16_ H_28_ O_3_			7.5060
Methyl 9,10-octadecadienoate (Methyl linoleate; C18:2)	C_19_ H_34_ O_2_	13.0068		14.9364
Cyclooctasiloxane, hexadecamethyl-	C_16_ H_48_ O_8_ Si_8_		1.1240	
Cyclononasiloxane, octadecamethyl-	C_18_ H_54_ O_9_ Si_9_		1.1560	
n-Hexadecanoic acid (palmitic acid)	C_16_ H_32_ O_2_	18.0802	6.25117	14.6338
Cyclononasiloxane, octadecamethyl-	C_18_ H_54_ O_9_ Si_9_		0.8183	
Methyl 12,13-tetradecadienoate	C_15_ H_26_ O_2_		6.4013	4.6444
2-Methylcyclohexyl ethylphosphonochloridate	C_9_ H_18_ Cl O_2_ P		2.9027	
Myristoleic acid	C_14_ H_26_ O_2_		7.4220	
12-hydroxy-9-octadecenoic acid (Ricinoleic Acid,18:1-OH)	C_18_ H_34_ O_3_		71.9933	
cis-9-Tetradecenoic acid, isobutyl ester	C_18_ H_34_ O_2_		0.9368	
Heptasiloxane, 1,1,3,3,5,5,7,7,9,9,11,11,13,13-tetradecamethyl-	C_14_ H_44_ O_6_ Si_7_		0.9945	

### 2.2 Experimental setup for engine testing

**Fig. 2:**
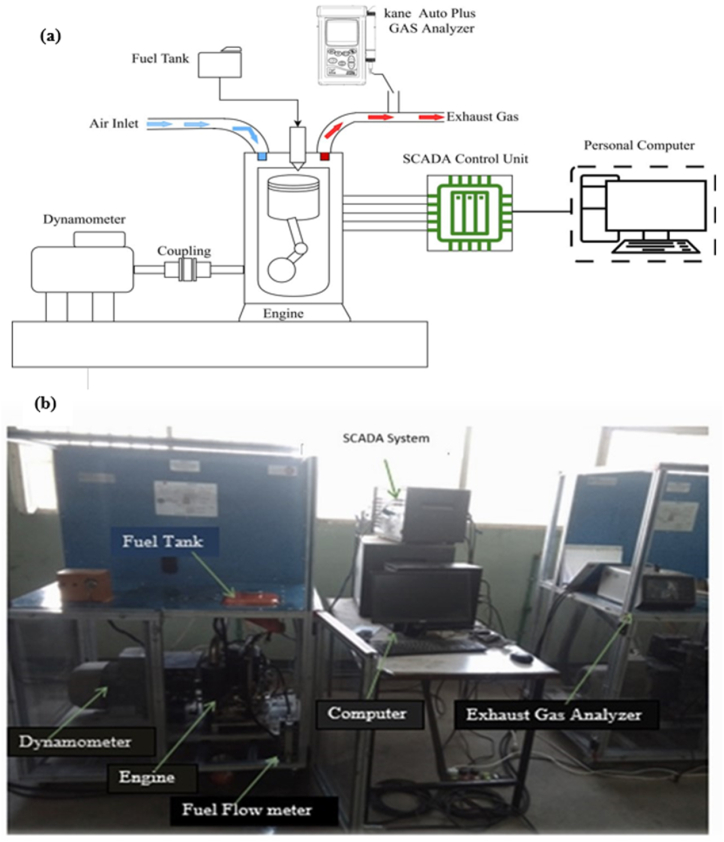
(a) Schematic layout engine setup and (b) Photographic view of engine setup (AASTU Lab).

The engine's performance was evaluated using the computer-controlled, 7.5 kW, single-cylinder, and four-stroke TBMC8 test bench (Fig. 2). Similarly, as shown in Fig. 2, the exhaust gas emission was examined using a Kane AUTO-plus five-gas analyzer. Exhaust emissions such as CO, CO2, HC, and NOx are evaluated along with engine performance indicators like braking torque, brake power, brake thermal efficiency, and specific fuel consumption. A 400 mL sample from each mix was added to the petrol tank, and the engine was operated for about 10 min. The SCADA software of the engine captured the engine's performance parameters. To burn off any fuel residue that might still be in the fuel line, the engine was run for five additional minutes after a test was completed, at which point the fuel was emptied and the next fuel blend was added to the fuel tank. The completed specification of the TBMC8 Computer Controlled Test Bench Engine is presented in Table 3.

**Table 3:** Specification of TBMC8 computer controlled test bench engine.

**Type**	**4-stroke, single cylinder**
Displacement	309 c.c.
Bore X Stroke	75 × 70 mm
Max. horsepower	7.5 kW/30000 rpm
Max. torque	18 Nm/2400 rpm
Dry weight	54 kg
Cooling system	Water-cooled (radiator)
Combustion system	Spherical type
Fuel	SAE No.2-D light diesel oil
Lubricating system	Forced lubrication with trochoid pump
Rotational direction	Counterclockwise facing flywheel
Cooling water capacity	1.2 L
Engine oil capacity	1.3 L
Starting system	Electric start
Starter	12 V 0.8kw

The test engine was ran until it reached operating temperature before the engine's performance and exhaust emission data were assessed. The performance and exhaust emission values of fuels prepared with volumetric test engines, D100, D99.50 (COSO + CASO) 0.50, and D99 (COSO + CASO) 1, were measured while the engines were run at specific engine loads. Equations eq. 3, eq. 4 were used to compute the BSFC and BTE, respectively. Equation (2) was used to calculate the mass of fuel consumed by the engine under various operating conditions. Fuel consumption is expressed as mass flow per unit time (mass flow rate), and volume flow rate is usually given according to a relation that quantifies the amount of fuel consumed per unit time. The brake-specific energy consumption (BSFC), which may regulate both the mass flow rate and fuel heating value, is an essential engine parameter. Equation (3) below can be used to estimate the BSFC. Equation (4) was also used to determine the brake thermal efficiency $(\eta_{bth)}$) and the results were shown in the results and discussion section.

eq. 1 $${\dot{V}}_{f}=\frac{v_{f}}{t}$$

The density and the fuel volume are calculated using eq. (2) below. Where, $v_{f}$ - volume flow meter of fuel (m^3^/h) and mf = fuel consumption (kg/h)

eq. 2 $${\dot{m}}_{f}=mass\;flow\;rate=\frac{v_{f}*\rho_{f}}{t},where\;\rho_{f}=density\;of\;fuel$$

eq. 3 $$BSFC=\frac{{\dot{m}}_{f}}{Brake\;power\left(Bp\right)}$$

eq. 4 $$Brake\;thermal\;efficiency\left(BTE\right)=\frac{Brake\;power\;\left(kW\right)*3600}{{\dot{m}}_{f}\left(kg/h\right)*calorific\;value\;\left(kJ/kg\right)}*100\%$$

### 2.3 Uncertainty of measurement

Uncertainty in an experiment can arise from a number of factors, such as the kind of instruments utilized, the measurement technique, the surroundings, and the experimental configuration. After applying the various engine loads and various speed for 5 min in each scenario, engine performance and emissions were tested to make sure the measured values were constant. For every fuel, five runs of the experiment were made. To calculate the total uncertainty using detected parameters such brake torque, exhaust gas temperature, carbon monoxide, carbon dioxide, and nitrogen oxides, the root mean square of the uncertainty in the experimental data and the instrumental uncertainty was utilized [15]. In particular, y1, y2, y3, and so on are independent variables that have an impact on a certain function, *f(y)*. In particular, ×1, ×2, ×3, and so on are independent variables that have an impact on a certain function, F. Furthermore, the independent variable errors are w1, w2 … and wn, and wF stands for the overall percentage error. To ensure that the test findings were accurate, an error analysis was performed using Taylor's theorem. By doing the measurement three times, the overall measurement uncertainty for those variables is less than 5 % standard deviation. Equation (5) was used to get the overall degree of uncertainty, which is displayed in Table 4.

eq. 5 $$\begin{array}{l} Overall\;uncer\;\tan\;ity=\\ (\sqrt{{\left(\Delta BP\right)}^{2}+{\left(\Delta BT\right)}^{2}+{\left(\Delta BTE\right)}^{2}+{\left(\Delta BSFC\right)}^{2}+{\left(\Delta EGT\right)}^{2}+{\left(\Delta CO\right)}^{2}+{\left(\Delta CO_{2}\right)}^{2}} \end{array}$$

**Table 4:** Uncertainty values for various combustion parameters.

Parameter	Range	Accuracy	uncertainty
Engine speed	1–3250	±0.202	±0.25
Brake torque (BT)	–	±105 Nm	±1.3
Brake power (BP)	–	±0.032 KW	±0.3
Brake thermal efficiency (BTE)	–	±0.501 %	±1.7
exhaust gas temperature (EGT)	30–1000	±4.89	±1.9
Carbon monoxide	0–10 %	±0.0605 %	±0.56
Carbon dioxide	0–15 %	±0.5	±1
Carbon monoxide	0–15 %	±0.058	±0.22

## 3 Results and discussions

The effects of adding blended seed oil made of cotton and castor to diesel fuel are examined in terms of emissions and engine performance using a computer-controlled, 7.5 kW, single-cylinder, four-stroke TBMC8 test bench. Three fuel samples were used to feed the test engine: D100, a commercial diesel fuel; D99.50 % (COSO + CASO) 0.5 and D99 (COSO + CASO) 1, which were blends of cotton and castor seed oil with commercial diesel fuel at volumetric ratios of 0.5 % and 1 %. Exhaust emissions (including NOX, CO2, CO, and HC) and engine characteristics like fuel and energy consumption, effective thermal efficiency, and others were studied. Each test was conducted under various speed conditions and with a distinct engine load. The test engine operated as shown in Fig. 2, increasing engine speed when the dynamometer load decreased or vice versa, thanks to the counter electromagnetic force that was supplied.

### 3.1 Performance characteristics

**Fig. 3:**
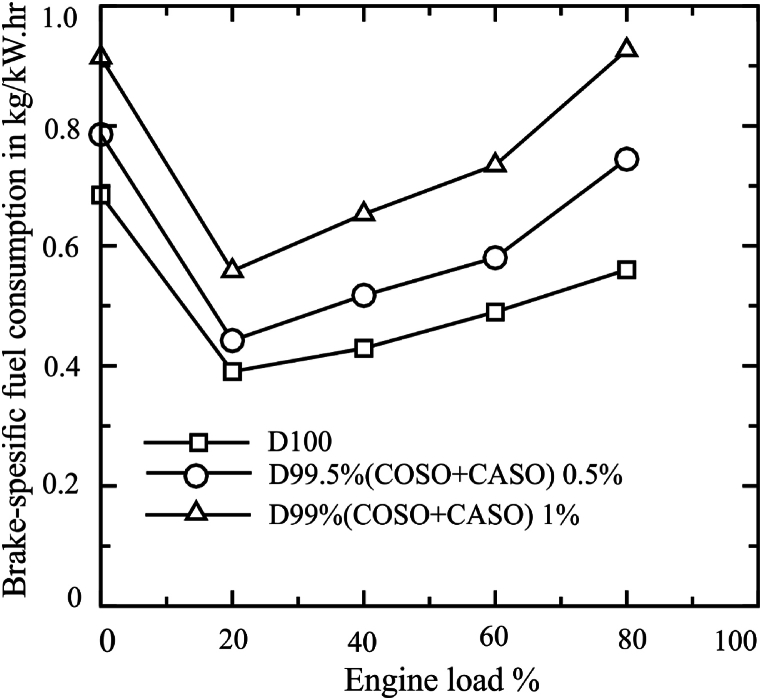
Braking-specific fuel consumption (BSFC) of blended fuels under loads of 20 %, 40 %, 60 and 80 %.

The performance of the combustion is examined using the TBMC8 test bench. The engine power, torque, brake-specific fuel consumption, and brake thermal efficiency (BTE) are obtained by adjusting the engine load conditions for 0 %, 20 %, 40 %, 60 %, and 80 %. The fuel consumption and power output are derived from the brake-specific fuel consumption (BSFC). It is an essential consideration when selecting the proper fuel because it shows the diesel engine's fuel efficiency and economy [21]. The brake-specific fuel consumption (BSFC) of the engine for three different test fuel types, corresponding to different engine running loads, is shown in Fig. 3. The findings demonstrated how the engine load affects the diesel brake-specific fuel consumption (BSFC).

The three fuel samples D100, D99.50 % (COSO + CASO) 0.5, and D99 (COSO + CASO) 1 were found to have the lowest BSFC at 20 % engine load, corresponding to 0.390, 0.442, and 0.558 kg/kWh, respectively. Similarly, the average brake-specific fuel consumption (BSFC) values of D100, D99.50 % (COSO + CASO) 0.5, and D99 (COSO + CASO) 1 were 0.511, 0.614, and 0.757 kg/kWh, respectively. Accordingly, it was shown that the total BSFC gain from diesel fuel was 20.16 % better for D99.50 % (COSO + CASO) 0.5 and 48.14 % better for D99 (COSO + CASO) 1. Brake-specific fuel consumption (BSFC) is directly impacted by fuel characteristics such as fuel density, viscosity, and calorific value (CV). According to earlier studies, employing biodiesel results in a higher brake-specific fuel consumption (BSFC) for the same power output than diesel [[22], [23], [24]]. This is because biodiesel has a lower heating value than diesel [25,26].

### 3.2 Brake thermal efficiency

**Fig. 4:**
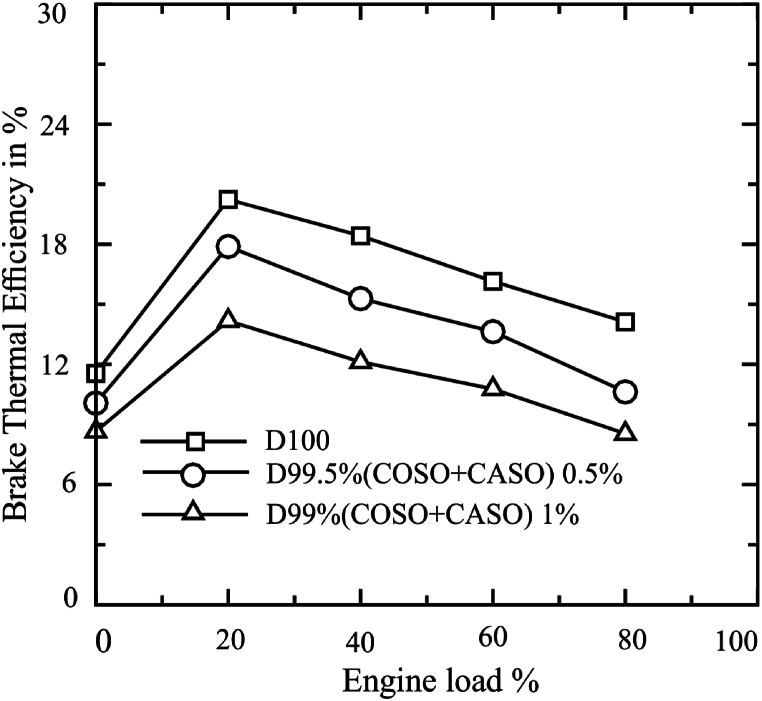
Brake thermal efficiency (BTE) of blended fuels under loads of 0 %, 20 %, 40 %, 60 and 80 %.

The diesel fuel's brake thermal efficiency (BTE) variation with engine load for the 0.5 % and 1 % bio-additives is shown in Fig. 4. All fuels under examination had the highest brake thermal efficiency at 20 % engine load and the brake thermal efficiency declined slightly as engine load decrease from 20 % to 80 %. At 20 % engine load, the highest BTE values for D100, D99.50 % (COSO + CASO) 0.5, and D99 (COSO + CASO) 1 were 20.24 %, 17.89 %, and 14.18 %, respectively. The Brake thermal efficiency (BTE) were 16.19 %, 13.50 %, and 10.85 % for D100, D99.50 % (COSO + CASO) 0.5, and D99 (COSO + CASO) 1. For D99.50 % (COSO + CASO) 0.5 and D99 (COSO + CASO) 1, the overall BTE was found to be 16.62 % and 32.98 % lower than diesel fuel, respectively.

Due to its higher viscosity, lower calorific valve, higher density, diesel fuel with bio-additive has a lower BTE than regular diesel fuel. Better atomization, finer droplets, and a higher calorific value demonstrate the higher BTE for the fuel content of petro diesel. Biodiesel burns more unevenly than diesel fuel because higher viscosity reduces fuel vaporization and atomization [[27], [28], [29]]. Simsek and Uslu's [30] work used RSM to determine the ideal operating conditions for compression ignition engines running on biodiesel/EHN fuel mixtures in terms of both exhaust emissions and optimal performance. Diesel with bio-additives in the current study, however, has an acceptable range for brake thermal efficiency at maximum engine speed.

### 3.3 Combustion efficiency characteristics

**Fig. 5:**
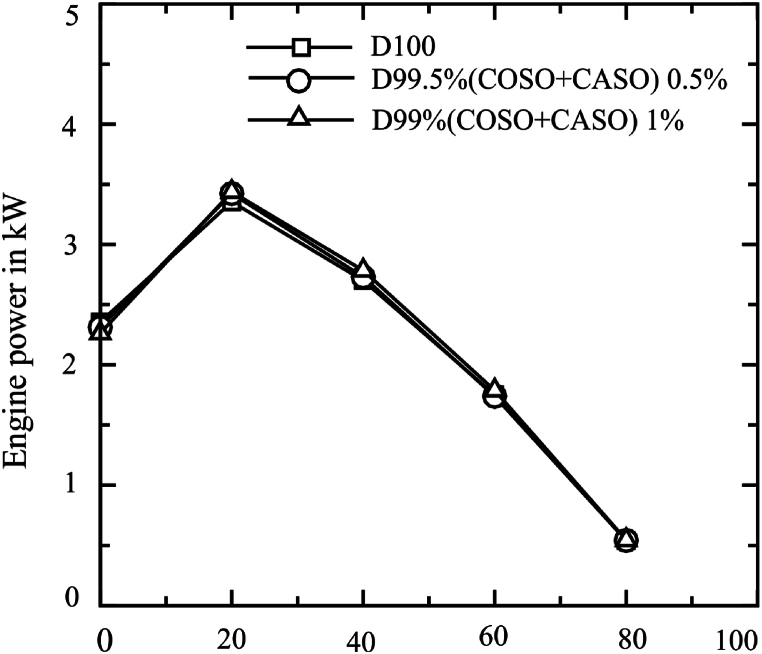
Engine power of blended fuels under loads of 0 %, 20 %, 40 %, 60 % and 80 %.

Fig. 5 shows the relationship between engine load variation and engine power for each of the three fuels that are being studied. The highest engine power is recorded in all three scenarios at 20 % engine load and is progressively reduced when engine load varies from 20 % to 80 %. The highest engine power values at 20 % engine load were 3.355, 3.423, and 3.438 KW for D100, D99.50 % (COSO + CASO) 0.5, and D99 (COSO + CASO) 1, respectively. The average engine power values were 2.1402, 2.149, and 2.1632 KW for D100, D99.50 % (COSO + CASO) 0.5, and D99 (COSO + CASO) 1, respectively. The higher oxygen content in biodiesel blends, which promotes more complete combustion, was the main factor in the increased engine power for D99.50 % (COSO + CASO) 0.5 and D99 (COSO + CASO) 1, which were found to be 0.4 % and 1.1 % higher than diesel fuel, respectively. The high lubricity of biodiesel results in less friction loss and so improves brake power [31,32]. In addition, because biodiesel mixtures have a higher density than pure diesel, a higher fuel flow rate must be injected into the engine cylinder in order to maintain the same fuel volume [33].

**Fig. 6:**
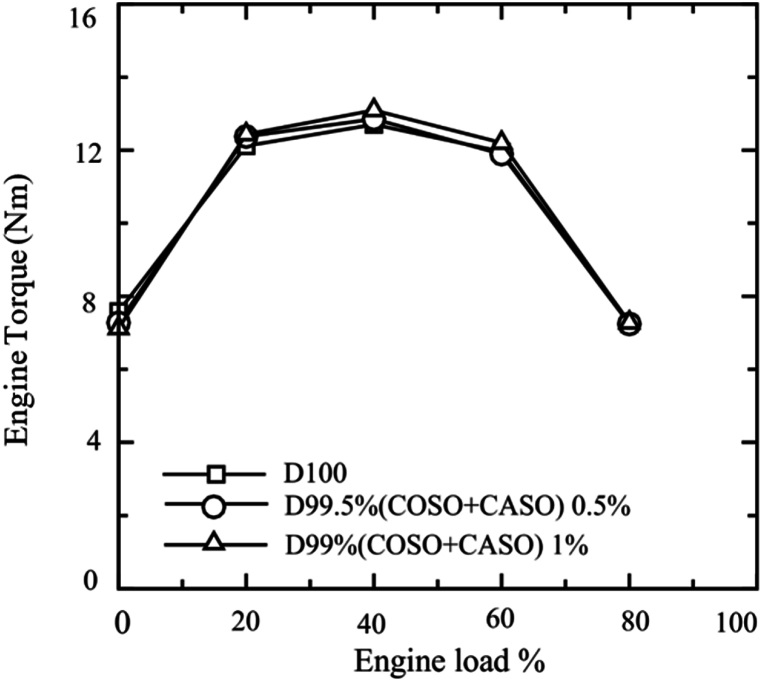
Engine torque of blended fuels under loads of 0 %, 20 %, 40 %, 60 % and 80 %.

The relationship between engine torque and engine load for each of the three fuel scenarios is shown in Fig. 6. At 40 % engine load, the highest torque were recorded for all the three fuels. The engine's torques decreased mostly because of a decrease in volumetric efficiency following the engine load rise [2]. At 40 % engine load, the highest engine torque values for D100, D99.50 % (COSO + CASO) 0.5, and D99 (COSO + CASO) 1 were 12.704, 12.855, and 13.096 Nm, respectively. The average engine torque values were 10.32, 10.33, and 10.43 Nm for D100, D99.50 % (COSO + CASO) 0.5, and D99 (COSO + CASO) 1, respectively. It was found that the overall engine torque increase for D99.50 % (COSO + CASO) 0.5 and D99 (COSO + CASO) 1 was 1.1 % and 0.1 % higher than diesel fuel, respectively. This is because of the increased oxygen content in the combination of castor seed and cottonseed oil (COSO + CASO), which dominates the oil's higher viscosity and density and results in the phenomena of total combustion. The average pressure inside the engine cylinder will rise as a result, increasing power as well as brake torque and piston force. Other researcher reported similar result [34].

### 3.4 Emission characteristics

**Fig. 7:**
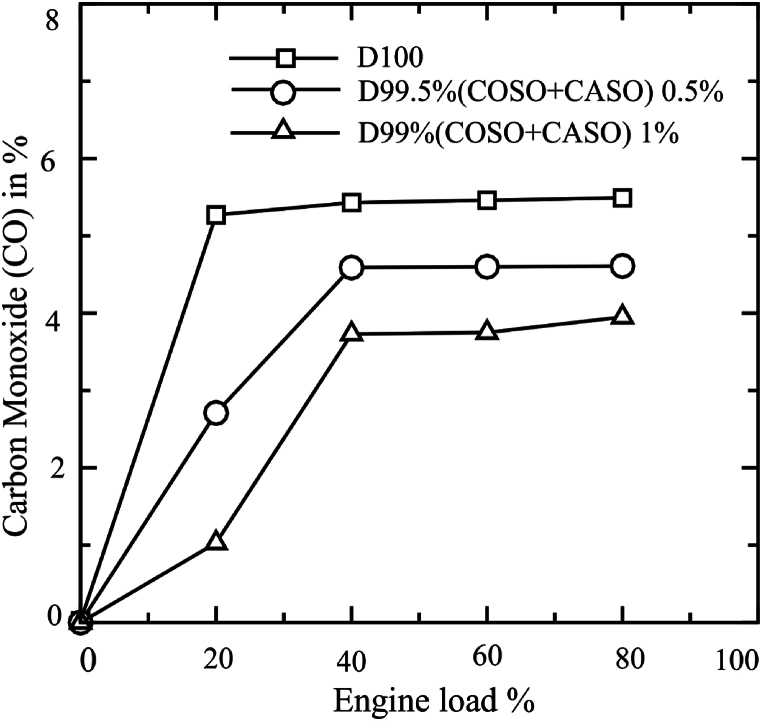
Carbon monoxide (CO) emissions of blended fuels under loads of 0 %, 20 %, 40 %, 60 % and 80 %.

A number of researchers are attempting to lower harmful emissions from engines. The experimental results on the exhaust emission parameters using diesel fuel, D99.50 % (COSO + CASO) 0.50 % and D99 % (COSO + CASO) 1 %, are presented and discussed in this section. Carbon monoxide (CO) is created because of incomplete combustion and partial oxidation of the fuel's carbon atoms. Temperature and the engine's air-to-fuel ratio are two important parameters that affect incomplete combustion, which causes CO emissions [33]. The Carbon monoxide (CO) emissions of three different fuel samples—D100, D99.50 % (COSO + CASO) 0.5, and D99 (COSO + CASO) 1—under various engine loading conditions are presented in Fig. 7. D99 (COSO + CASO) 1, D99.50 % (COSO + CASO) 0.5, and D100 had average CO emissions of 2.49 %, 3.30 %, and 4.33 %, respectively. In comparison to diesel fuel, the total CO emissions reductions for D99.50 % (COSO + CASO) 0.5 and D99 (COSO + CASO) 1 were found to be, respectively, 23.79 % and 42.49 % lower. When bio-additives are used, the amount of CO emissions decreases significantly. Using biodiesel leads to lower CO emission values due to the presence of the higher oxygen content. This is because more CO oxidation during engine exhaust is encouraged by the higher oxygen level. Numerous research on the emissions of biodiesel have produced results that support this output [[35], [36], [37], [38]]. As a result, higher oxygen concentrations promote full burning, which reduces hydrocarbon (HC) content to zero. Depending on the feedstock oil or fat utilized, the final biodiesel will have a different position in the trade-off between cetane number, oxidation stability, and cold flow ability. Higher cetane levels and lower cold flow characteristics are observed in biodiesel produced from more saturated feedstock, likely due to enhanced oxidation stability. The cold flow properties of biodiesel produced from feedstock oil or fat with low saturated content are superior, despite its lower cetane number and worse oxidation stability. Caster seed oil's higher ricinoleic acid (18:1-OH) content was mixed with cottonseed oil's higher polyunsaturated content to obtain the optimum bio additive properties [[39], [40], [41]].

**Fig. 8:**
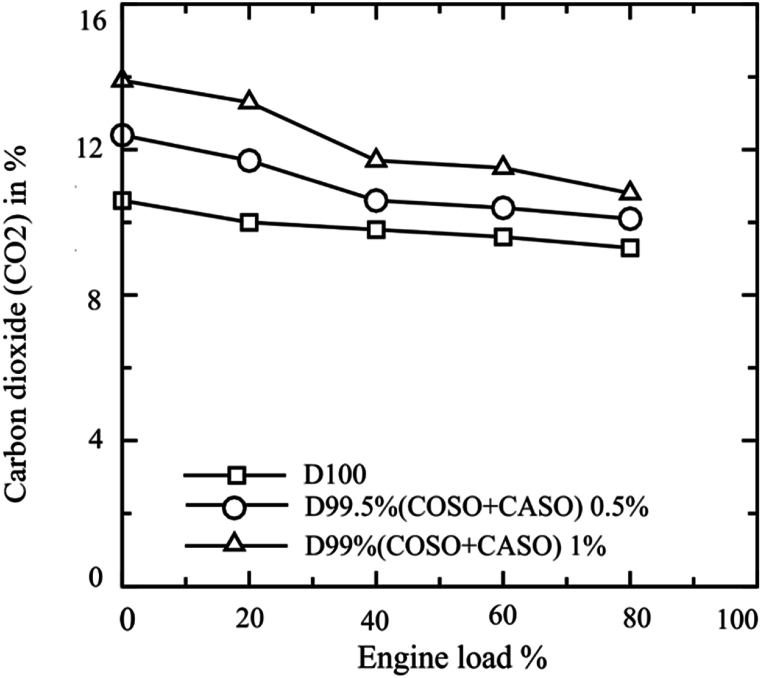
Carbon dioxide (CO2) % emissions of blended fuels under loads of 20 %, 40 %, 60 % and 80 %.

The amount of CO2 emitted by the engine at various engine loads for all testing fuels is shown in Fig. 8. According to Fig. 8, CO2 generation dropped with increasing engine load for all fuels under investigation. For the D100, COSO + CASO 0.5, and D99 (COSO + CASO) 1, the average CO2 emissions were 9.86 %, 11.04 %, and 12.24 %, respectively. For a diesel with bio-additives, the average CO2 emissions were 11.97 % and 24.14 % higher, respectively, for D99.50 % (COSO + CASO) 0.5 and D99 (COSO + CASO) 1. The increased oxygen content in the blended fuels ensured more conversion from CO to CO2 by enhancing complete combustion.

**Fig. 9:**
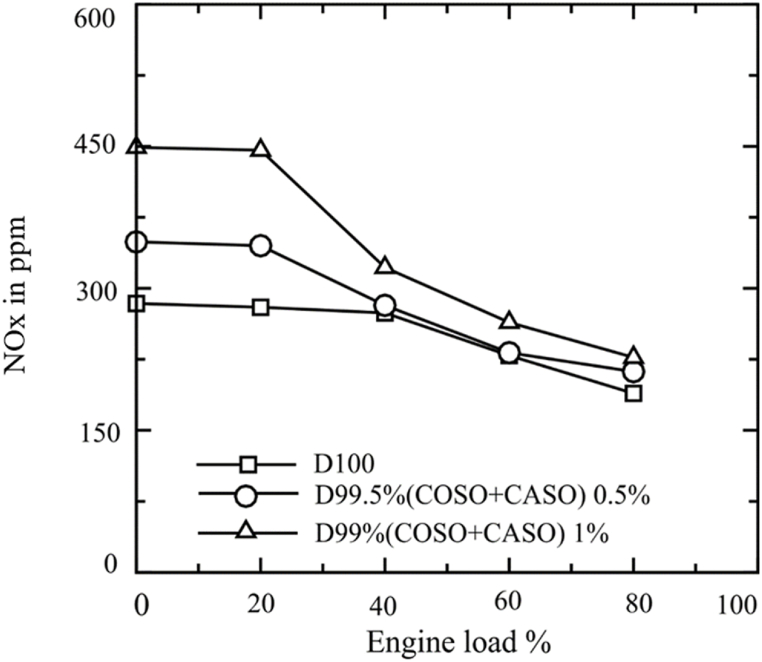
NOx emissions of blended fuels under loads of 20 %, 40 %, 60 % and 80 %.

The amount of nitrogen oxides produced in the combustion chamber can be affected by a number of factors, including temperature, air volume, cylinder geometry, intake air temperature, pressure, fuel characteristics, compression ratio, injection time, and injection pressure. All of the fuels under investigation's NOx emissions at various engine loads are presented in Fig. 9. The graph indicates a decrease in NOx generation with increased engine load for all tested fuels under the study. Average NOx Emissions: 251.2, 284, and 341.6 ppm were recorded for D100, D99.50 % (COSO + CASO) 0.5, and D99 (COSO + CASO) respectively. Accordingly, the average NOx emissions for D99 (COSO + CASO) 1 and D99.50 % (COSO + CASO) 0.5 were 36 % and 13.1 % higher than those of diesel fuel, respectively. Blends incorporating biodiesel are anticipated to burn more efficiently and fast due to the fuel's high CN content and short ignition delay.

## 4 Conclusions

Biodiesel is superior to petroleum diesel in many respects; however, the high cost of feedstock and the lack of economic and technical feasible always pose challenges to its efficient production from any kind of feedstock. The use of cotton and castor blend bio-additives to diesel fuel significantly improved the combustion and emission performance of the diesel. Therefore, the following conclusions were drawn from the results and discussions part.

✓ When compared to pure diesel fuel, the usage of bio-additives produced from a blend of cotton and castor resulted in a discernible increase in Brake Specific Fuel Consumption (BSFC). Their higher viscosity and lower calorific value, which affected the fuel's atomization and combustion efficiency, were the main causes of this. On the other hand, Brake Specific Fuel Consumption (BSFC) is within an acceptable range for engine loads between 20 % and 60 %.
✓ Because blended cotton and castor additives are more lubricating and have a larger oxygen content, they reduce friction losses and increase combustion efficiency in diesel. This results in a small improvement in engine power and torque. As a result, the brake thermal efficiency reaches its peak when the engine load is equal to 20 % and then gradually decreases as the engine load increases to 80 %.
✓ Due to the increased oxygen content of bio-additives, which aids in CO oxidization during combustion, carbon monoxide (CO) emissions have dropped by 1.5 % for engine loads ranging from 0 % to 80 %. It was demonstrated, however, that the higher carbon dioxide (CO2) emissions of biodiesel blends were caused by their elevated oxygen content, which promotes the conversion of CO to CO2.
✓ Nitrogen oxide (NOx) emissions slightly increased because of the engine's high temperature brought on by the blends of cotton and castor biodiesel additives. This was most likely brought on by the blends' high oxygen content, which allowed for quicker and more effective combustion, and high cetane number, which reduced the ignition delay.

## 5 Future work

The quality advantages of both oils can be utilized when combining cottonseed and castor seed oils as additives in diesel engines that run on low sulphur diesel fuel. This combination has a favorable fatty acid profile, and all of its physicochemical characteristics are within the ASTM requirements for diesel fuel. Extraction of cottonseed oil with less gossypol and without the use of dangerous chemicals is essential for research. To acquire cottonseed oil low or free gossypol content for their anticipated purposes in the future is the focus of several research studies.

## CRediT authorship contribution statement

**Hailegebrel Zewdie Woldetensy:** Investigation, Formal analysis, Data curation. **Dinku Seyoum Zeleke:** Supervision, Methodology, Conceptualization. **Getachew Shunki Tibba:** Writing – review & editing, Resources, Data curation.

## Data availability

Article-cited, added content, or material mentioned in the article. Furthermore, the data is accessible upon request.

## Declaration of competing interest

The authors whose names are listed immediately below certify that they have NO affiliations with or involvement in any organization or entity with any financial interest (such as honoraria; educational grants; participation in speakers’ bureaus; membership, employment, consultancies, stock ownership, or other equity interest; and expert testimony or patent-licensing arrangements), or non-financial interest (such as personal or professional relationships, affiliations, knowledge or Beliefs) in the subject matter or materials discussed in this manuscript.
